# *De novo* genome assembly of Texas wintergrass [*Nassella leucotricha* {Trin. & Rupr.} Pohl] supports diagnostic marker development and phylogenetic analysis of species within the family Poaceae

**DOI:** 10.1093/g3journal/jkag225

**Published:** 2026-08-19

**Authors:** Christian J Stephens, Nathaniel Jacob Turner, Puneet Kaur Mangat, Avinash Shrestha, Md Monjurul Huda, Rosalyn B Angeles-Shim

**Affiliations:** Department of Plant and Soil Science, Davis College of Agricultural Sciences and Natural Resources, Texas Tech University, Lubbock, TX 79401, United States; Department of Plant and Soil Science, Davis College of Agricultural Sciences and Natural Resources, Texas Tech University, Lubbock, TX 79401, United States; Department of Plant and Soil Science, Davis College of Agricultural Sciences and Natural Resources, Texas Tech University, Lubbock, TX 79401, United States; Department of Plant and Soil Science, Davis College of Agricultural Sciences and Natural Resources, Texas Tech University, Lubbock, TX 79401, United States; Department of Plant and Soil Science, Davis College of Agricultural Sciences and Natural Resources, Texas Tech University, Lubbock, TX 79401, United States; Department of Plant and Soil Science, Davis College of Agricultural Sciences and Natural Resources, Texas Tech University, Lubbock, TX 79401, United States

**Keywords:** native forages, marker resource, HiFi, genome assembly

## Abstract

Texas wintergrass is a cool-season perennial bunchgrass native to North America with ecological and agronomic importance as a winter forage species, yet genomic resources for this species remain limited. Here, we present the first de novo genome assembly of *Nassella leucotricha* generated using PacBio HiFi sequencing. Assembly with hifiasm produced a 959.92-Mb genome with 8× coverage, comprising 2,394 contigs with an N50 of 530.6 kb. Assembly completeness was high, with 99.6% of Benchmarking Universal Single-Copy Orthologs identified, although a substantial proportion were duplicated. Genome size estimates based on flow cytometry and k-mer analysis, together with assembly metrics, indicate a repeat-rich and structurally complex genome. Repeat annotation revealed that 64.42% of the genome consists of repetitive elements, predominantly long terminal repeat retrotransposons, including Ty1/Copia and Gypsy/DIRS1. Gene prediction using the homology-based pipeline GeMoMa identified 54,132 high-confidence genes, of which 53,868 were functionally annotated and 26,346 were assigned to KEGG pathways. Mining for genome-wide simple sequence repeats identified over 38,000 markers, with a validated subset demonstrating use for genetic diversity analysis. Reference-guided alignment and scaffolding using *Brachypodium distachyon* and *Oryza sativa* provided comparative frameworks for evaluating conserved sequence relationships between Texas wintergrass and representative grass genomes. Our results establish a genomic resource for Texas wintergrass, supporting future studies in comparative genomics and molecular breeding.

## Introduction

Texas wintergrass [*Nassella leucotricha* {Trin. & Rupr.} Pohl] is a cool-season perennial bunchgrass native to Texas (Van Auken 1997) (Fig. 1a and b). This small-seeded grass species holds economic and ecological significance as a winter forage due to its ability to remain green and palatable during the colder months, while warm-season grasses become dormant (Brown 1949; Van Auken 1997). Inherently, this makes Texas wintergrass a vital resource for sustaining livestock productivity in winter grazing systems (Sanders 1975; Angell et al. 1986; Pinchak et al. 1990).

**Fig. 1.**
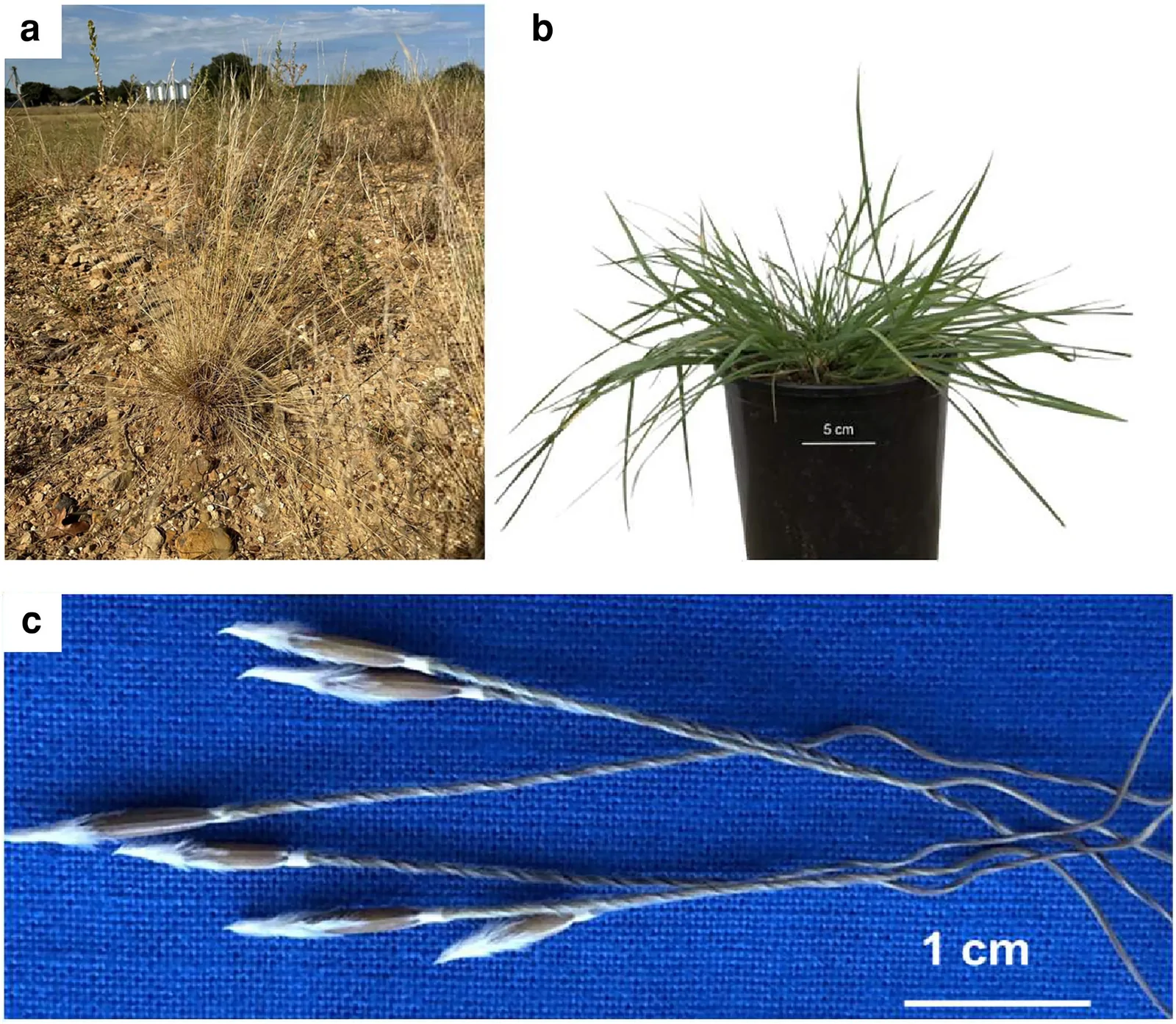
Morphology of Texas wintergrass (*N. leucotricha*). a) Naturalized population of Texas wintergrass growing on a rocky roadside in Breckenridge, Texas. b) Individual plant cultivated under greenhouse conditions in a 4-inch pot. c) Gross morphology of the caryopses (seeds) showing the characteristic barbs. Scale bars: 5 cm (b), 1 cm (c).

Despite its agronomic potential, Texas wintergrass remains underutilized as a commercial forage due to numerous physiological and morphological constraints. Primarily, its inconsistent seed germination complicates stand establishment and limits its scalability in managed pastures. Morphologically, traits such as barbed awns and brittle rachillas hinder its mechanical harvesting and seed handling (Fig. 1c). These features, while beneficial for natural dispersal, pose significant barriers to domestication and commercial seed production.

In recent studies, it was demonstrated that dormancy in Texas wintergrass is not absolute. Dormancy of the seed can be modulated through chemical scarification and exogenous application of low concentrations of hydrogen peroxide, promoting oxidative signaling and weakening the seed coat (Turner et al. 2024). While these findings offer short-term solutions to dormancy, they do not resolve the genetic limitations that restrict broader agronomic use of the species. Long-term improvement of Texas wintergrass requires both functional genetic analysis and breeding interventions. Traits such as seed dormancy, awn morphology, and seed shattering are all under genetic control. The modification of these traits through modern breeding could lead to more agronomically favorable cultivars (Guo et al. 2004; Konishi et al. 2006; Heffner et al. 2009). Molecular breeding tools, such as marker-assisted selection and genome editing, have proven effective in accelerating trait improvement by enhancing selection efficiency and reducing breeding cycle duration (Varshney et al. 2005a; Angeles-Shim et al. 2020; Reyes et al. 2021).

Whole-genome sequencing supports many downstream applications, including transcriptomics, proteomics, genome-wide association studies, and genome editing. These applications provide a powerful means to dissect complex traits, identify novel alleles, and accelerate domestication (Bernal-Gallardo and de Folter 2024). Sequencing efforts in orchard grass (*Dactylis glomerata*), weeping lovegrass (*Eragrostis curvula*), and perennial ryegrass (*Lolium perenne*) have revealed the genetic factors underlying flowering time, forage quality, and the evolutionary relationships between these grasses (Carballo et al. 2019; Huang et al. 2020; Frei et al. 2021).

Given its agronomic limitations, the development of a reference genome for Texas wintergrass is essential. Whole-genome assembly will provide the foundation of sequence information needed to identify loci associated with traits, such as seed dormancy, awn morphology, and forage quality. This study aims to generate the first whole-genome assembly of Texas wintergrass toward unlocking its potential as a commercially viable and ecologically resilient winter forage crop.

## Materials and methods

### Plant materials and DNA extraction

High molecular weight DNA was extracted from leaf tissue of Texas wintergrass using a modified CTAB protocol (Murray and Thompson 1980). The resulting DNA was treated with 2 µL of RNase (10 mg/mL) at 37 °C for 30 min to remove any residual RNA. DNA quality and quantity were assessed using a NanoDrop One Microvolume UV-Vis Spectrophotometer, ensuring the suitability of the DNA samples for downstream applications.

### Genome size estimation using flow cytometry

The genome size of Texas wintergrass was estimated with flow cytometry, using rice (*Oryza sativa*) as an internal standard. Rice was selected as a reference due to its well-characterized genome and established genetic relationship to other grass species (Kellogg 1998). Leaf samples from both rice and Texas wintergrass were cut into 2-cm segments and then placed in chilled Petri dishes containing 2 mL of lysis buffer (15-mM Tris, 2-mM Na_2_EDTA, 0.5-mM spermine, 80-mM KCl, 20-mM NaCl, 0.1% Triton X-100, pH 7.5, with 15-mM β-mercaptoethanol) (Dpooležel et al. 1989). Tissues were macerated on ice, supplemented with an additional 2 mL of buffer, and triturated gently. The resulting suspension was filtered through a 30-µm nylon mesh, and 1-mL aliquots were transferred to 2-mL tubes. One tube was stained with 50-µg/mL propidium iodide, and both were treated with 50-µg RNase. Samples were incubated for 30 min at room temperature and stored at 4 °C until analysis. Flow cytometry was performed at the TTU College of Arts & Sciences Microscopy facility. Genome size was calculated using the formula (rice 1C peak/wintergrass 1C peak) × 0.5 pg, where C refers to the amount of DNA contained within a haploid nucleus (1C). The 1C value of rice (0.5 pg) was used as a reference to estimate the nuclear DNA content of Texas wintergrass. Final estimates were converted from picograms to megabases and averaged from 2C peak values.

### Genome size estimation and ploidy inferencing using Genomescope 2.0 and Smudgeplot

To further estimate genome size, heterozygosity, and ploidy, complementary k-mer-based approaches were applied using both Illumina short-read and PacBio HiFi long-read sequencing data. Genome size and heterozygosity were first estimated using GenomeScope2, which models k-mer frequency distributions derived from high-accuracy short reads (Ranallo-Benavidez et al. 2020). Illumina reads were used for this analysis due to their low sequencing error rate, which is imperative for accurate k-mer spectrum modeling. k-mer counts were generated using Jellyfish v2.2.10 with a k-mer size of 21, and histograms were constructed using the jellyfish histo function (Marcais and Kingsford 2011). The resulting k-mer distribution was analyzed in GenomeScope2 to infer genome size, repeat content, and heterozygosity.

Ploidy and allelic structure were further evaluated using Smudgeplot v0.5.3, which leverages k-mer pair relationships to infer genome composition (Ranallo-Benavidez et al. 2020). For this analysis, PacBio HiFi reads were used to capture longer haplotypic context and improve detection of allele-specific k-mer pairs. k-mer tables were generated using FastK (k = 31), followed by extraction of heterozygous k-mer pairs using Smudgeplot hetmers with a lower coverage threshold (L = 40). The final ploidy visualization was produced using Smudgeplot all.

### Whole-genome sequencing, de novo assembly, and reference-guided scaffolding

For Illumina sequencing, the extracted DNA was sent to Novogene's sequencing facility in Sacramento, California, for DNA sample quality control. Once DNA was confirmed to be of suitable quality, a DNA library for the whole genome was prepared and used for whole-genome sequencing using a NovaSeq PE150 sequencer.

For PacBio Sequencing, DNA of Texas wintergrass was sent to Azenta Life Sciences (South Plainfield, NJ) for library preparation and whole-genome sequencing using the PacBio Sequel II platform. High molecular weight DNA was sequenced using the PacBio Sequel II platform, generating circular consensus sequencing (CCS) HiFi reads. De novo genome assembly was performed using hifiasm, a graph-based assembler optimized for HiFi reads, with default parameters and multithreading enabled (-t 32) (Cheng et al. 2021). Primary contigs were extracted from the assembly graph (.gfa) using custom AWK scripts to generate a FASTA-formatted assembly. Hifiasm's built-in duplication purging module was utilized to reduce redundancy arising from heterozygosity. Assembly statistics, including total assembly size, contig number, and N50, were calculated using SeqKit and custom scripts. Assembly completeness was assessed using Benchmarking Universal Single-Copy Orthologs (BUSCO) v6.0.0 with the embryophyta_odb10 dataset in genome mode (Simão et al. 2015).

### Reference-guided assembly reveals relatedness to clades in Poaceae

Reference-guided scaffolding was conducted using a minimap2 + RagTag pipeline, replacing earlier short-read alignment approaches in accordance with current best practices and reviewer recommendations (Li 2018; Alonge et al. 2021). Assembly-to-reference alignments were performed using minimap2 with the asm20 preset, which is optimized for divergent genome assemblies. Two reference genomes were used: *Brachypodium distachyon* v3.0 and rice (AGIS1.0 assembly; NCBI Assembly GCA_034140825.1) (International Brachypodium Initiative 2010; Shang et al. 2023). Scaffolding was performed using RagTag, which orders and orients contigs based on alignment to the reference genome. Assembly improvements were evaluated using QUAST and BUSCO, and alignment coverage was quantified from PAF files using custom AWK scripts to determine the proportion of the assembly aligning to each reference. Additionally, alignment to the wheat (IWGSC RefSeq v2.1; NCBI Assembly GCF_018294505.1) reference genome was performed using minimap2 to assess broader phylogenetic correspondence, though this was not used for scaffolding due to limited alignment coverage (International Wheat Genome Sequencing Consortium 2018).

### Assembly quality assessment and read mapping

To evaluate read support and assembly accuracy, HiFi reads were aligned back to the assembled genome using minimap2 with the map-hifi preset. Alignments were processed using SAMtools, including sorting and indexing of BAM files, followed by the generation of mapping statistics using samtools flagstat (Danecek et al. 2021). Assembly contiguity and quality metrics were further assessed using QUAST, allowing comparison between de novo and reference-guided assemblies (Gurevich et al. 2013). Postassembly redundancy was assessed using “purge_dups” to evaluate the contribution of residual haplotypic duplication to assembly size and BUSCO duplication (Guan et al. 2020). Assembly statistics and BUSCO completeness were then recalculated for comparison with the original hifiasm primary assembly generated using get_seqs.

### Repeat identification and genome masking

Repetitive elements were identified using a de novo repeat annotation pipeline consisting of RepeatModeler, RepeatClassifier, and RepeatMasker (Smit et al. 2008, 2015). A species-specific repeat database was built from the Texas wintergrass de novo assembly using BuildDatabase. RepeatModeler was then run with the NCBI search engine to identify repeat families directly from the genome assembly. The resulting consensus repeat library was classified using RepeatClassifier to generate a classified de novo repeat library for Texas wintergrass.

The classified RepeatModeler library was subsequently used as input for RepeatMasker to annotate and mask repetitive elements across the genome. RepeatMasker was run in sensitive mode with soft masking enabled (-s -xsmall), and GFF output was generated (-gff). This approach allowed for repetitive regions to be annotated while retaining the original nucleotide sequence in lowercase for downstream analyses. Repeat composition statistics were calculated from the RepeatMasker summary output to quantify the abundance of major repeat classes.

### Structural gene annotation using homology-based prediction

Gene prediction was conducted using GeMoMa. This homology-based gene prediction pipeline incorporates protein-coding gene models from related species (Keilwagen et al. 2019). Reference annotations from *O. sativa* and *B. distachyon* were downloaded from NCBI and used as the input for the GeMoMa pipeline. The GeMoMa pipeline was executed with multithreading (32 threads) and included alignment-based prediction, exon refinement, and gene model scoring. Output included predicted gene structures (GFF), coding sequences (CDSs), and translated protein sequences. Gene model completeness was assessed using BUSCO in protein mode with the *embryophyta_odb10* dataset.

Functional annotation of predicted protein sequences was performed using eggNOG-mapper, which assigns orthology, Gene Ontology (GO) terms, and KEGG pathways based on evolutionary relationships (Huerta-Cepas et al. 2019). The eggNOG database was locally installed, and annotation was performed using the emapper.py pipeline with multithreading enabled. Annotated outputs were used to quantify functional gene categories and assess annotation completeness.

### Phylogenomic analysis using single-copy BUSCO orthologs

To generate a sequence-based phylogeny of Texas wintergrass within Poaceae, predicted protein sequences from the species and representative published grass genomes were analyzed using BUSCO v6.0.0. The included taxa were Texas wintergrass, rice, *B. distachyon*, barley (*Hordeum vulgare*), perennial ryegrass, *Setaria italica*, sorghum (*Sorghum bicolor*), and corn (*Zea mays*). The Texas wintergrass protein set was derived from the GeMoMa-predicted annotation, while published proteomes for the remaining taxa were downloaded from NCBI using the NCBI Datasets command-line interface.

BUSCO was run independently for each species in protein mode using the *embryophyta_odb10* lineage dataset. Complete single-copy BUSCO sequences were extracted from each species, and only BUSCO orthologs present as single-copy genes across all included taxa were retained for phylogenetic analysis. Orthologous protein sequences were aligned individually using MAFFT, trimmed using trimAl, and concatenated into a single supermatrix. Maximum-likelihood phylogenetic inference was performed using IQ-TREE with automatic model selection. Branch support was assessed using 1,000 ultrafast bootstrap replicates and SH-aLRT support values. The resulting tree was visualized in R using the ape package.

### Simple sequence repeat marker development and validation

Simple sequence repeats (SSRs) were identified in the Texas wintergrass genome using the Genome-wide Microsatellite Analyzing Toward Application (GMATA) (Wang and Wang 2016), targeting motifs ranging from 2 to 10 bp. Primers were designed using the Primer3 tool integrated into the GMATA software. SSR mining was performed using GMATA on the masked Texas wintergrass assembly available at the time of marker development. The resulting SSR loci were used for primer design and validation, whereas the current de novo assembly, repeat annotation, and gene prediction statistics are reported separately from the revised hifiasm-based assembly.

A subset of 78 SSR markers were selected for validation via PCR using genomic DNA extracted from a population of Texas wintergrass sampled from Breckenridge, Texas. DNA was isolated from leaf tissue using a modified CTAB method (Murray and Thompson 1980). PCR amplification followed a standard protocol (Shim et al. 2015), with PCR conditions including an initial denaturation at 95 °C for 5 min, followed by 36 cycles of denaturation (95 °C), annealing (57 °C), and extension (72 °C) for 30 s each, and a final extension at 72 °C for 7 min. Amplicons were resolved on 3% agarose gels in 1× Tris-borate EDTA (TBE) buffer and visualized under UV light using a gel documentation system.

### Cross-species marker transferability and phylogenetic analysis

The transferability of 43 Texas wintergrass-specific SSR markers to other members of the family Poaceae, including rice, Eastern gamagrass (*Tripsacum dactyloides*), foxtail millet (*S. italica*), little barley (*Hordeum pusillum*), *Digitaria ciliaris*, and *B. distachyon*, was determined. These markers, which are distributed across the Texas wintergrass genome, were tested along with 4 rice-specific SSR markers. Leaf tissues were sampled from 21-d-old seedlings and stored in 2-mL microcentrifuge tubes. Genomic DNA was extracted using the TPS (100 mM Tris-HCl, pH 8.0, 1-M KCl, 10-mM EDTA) method (Konishi et al. 2006).

PCR was performed as described in the previous section, with the annealing temperature adjusted to 60 °C to enhance amplification specificity. Amplicons were resolved on 3% agarose gels in 1× TBE buffer and visualized under UV light.

Descriptive statistics for SSR markers, including the number of different alleles (Na), effective number of alleles (Ne), and expected heterozygosity (He), were computed in GenAlEx v6.5 within Excel (Peakall and Smouse 2012). Definitions and calculations follow the GenAlEx user guide (Appendix 1). Polymorphism information content (PIC) was calculated per locus using the formula PIC = 1 − ∑*p_i_^2^*, where *p^i^* is the frequency of the *i^th^* allele (Lemos et al. 2020). For loci scored as dominant (presence/absence) across taxa, PIC was computed as PIC = 2*p* (1 − *p*) with a theoretical maximum of 0.5, where *p* is the frequency of the band-present state.

Binary (+/−) scoring of the amplified bands was conducted and used to generate a pairwise genetic similarity matrix. A UPGMA dendrogram was constructed based on a simple matching dissimilarity matrix (Peakall and Smouse 2006). Tree construction and visualization were performed using iTOL v7 (https://itol.embl.de/). All analyses were conducted in RStudio (version 4.4.2) using the ape, dplyr, readr, and factoextra packages. Node support was evaluated using 10,000 bootstrap replicates.

## Results and discussion

### Genome size estimation and ploidy inference using k-mer analysis

Flow cytometric analysis of Texas wintergrass revealed distinct fluorescence peaks corresponding to both 1C and 2C nuclear DNA contents. Specifically, 1C peaks were observed at fluorescence intensities of 1491, 1010, and 2171, while 2C peaks were recorded at 3859, 2403, and 3952. For comparison, the internal standard species, rice, exhibited 1C peaks at 776, 535, and 1003 and 2C peaks at 1532, 1021, and 1667 (Fig. 2). Based on these measurements, the estimated genome size of Texas wintergrass averaged 776.58 Mb from 1C peaks and 941.62 Mbp from 2C peaks, resulting in a final genome size estimate of 859.1 Mb.

**Fig. 2.**
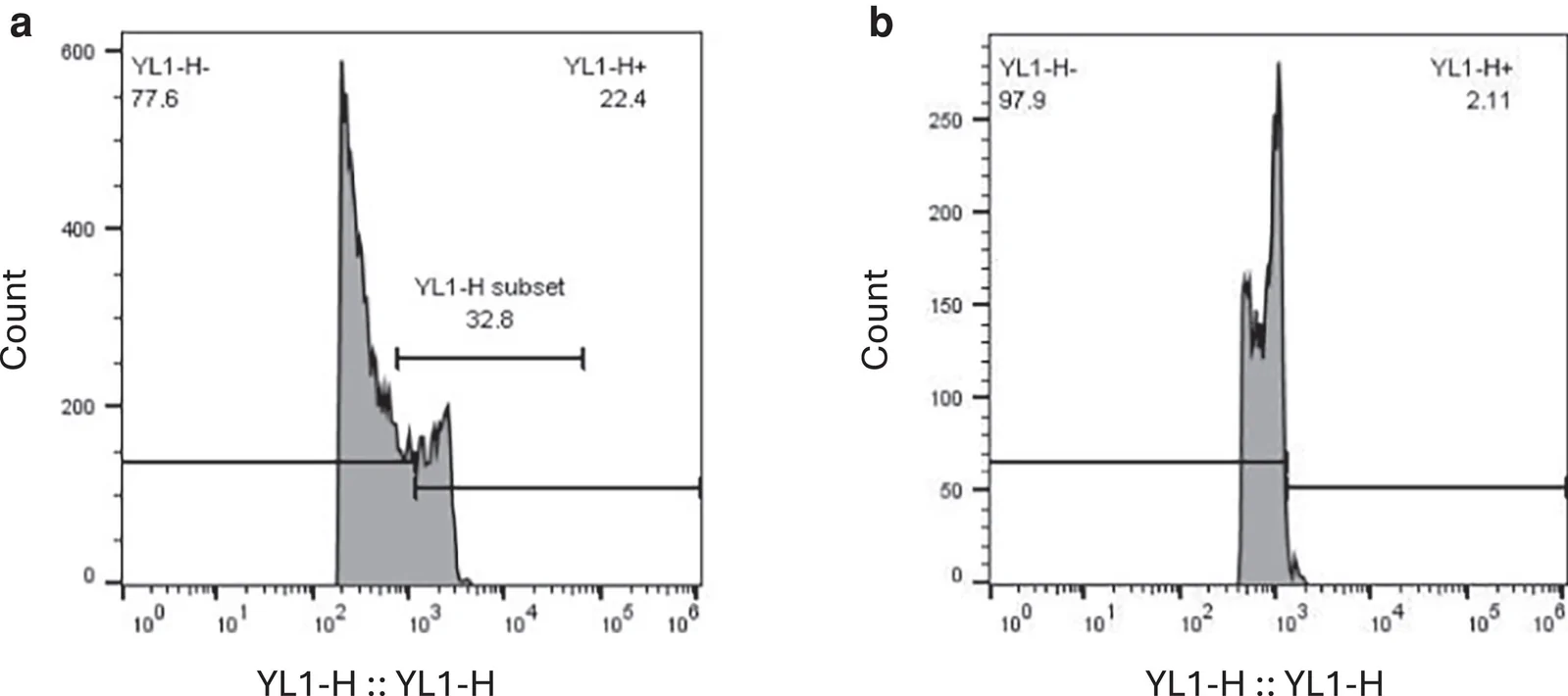
Flow cytometric estimation of nuclear DNA content in Texas wintergrass. Flow cytometry histograms showing relative fluorescence intensity corresponding to nuclear DNA content. The *x* axis represents fluorescence intensity (YL1-H), proportional to DNA content, and the *y* axis indicates nuclei count. a) Histogram of *N. leucotricha* nuclei showing a broader distribution of fluorescence intensity, with the primary peak corresponding to the G_0_/G_1_ phase and a secondary distribution representing higher DNA content nuclei. The gated region (YL1-H subset) indicates the population used for genome size estimation. The proportions of events within the negative (YL1-H−) and positive (YL1-H+) gates are indicated. b) Histogram of the internal reference standard used for calibration, displaying a narrow, well-defined peak corresponding to a known genome size. The tight distribution of the standard confirms instrument stability and provides a reference for converting fluorescence intensity to absolute genome size.

To complement flow cytometry, GenomeScope2 was performed using Illumina short reads, while Smudgeplot was performed using PacBio HiFi reads. GenomeScope2 is specifically designed for short-read sequencing data, as it relies on high-depth, low-error k-mer frequency distributions to model genome size, heterozygosity, and repeat content. In contrast, Smudgeplot leverages long-read-derived k-mer pair information to infer genome structure and ploidy, making it better suited for HiFi datasets despite their lower sequencing depth.

The GenomeScope2 profile based on 21-mers estimated a haploid genome size of approximately 406.2 Mb, with 43.7% unique sequence, a k-mer coverage peak of 23.7×, an estimated error rate of 0.267%, and heterozygosity of approximately 0.88% (Fig. 3a). The k-mer distribution showed a dominant peak at 24× coverage, having a low-coverage signal consistent with heterozygous k-mers. Additionally, we observed a subtle higher-coverage shoulder that may correspond to a collapsed or repetitive homozygous sequence. However, because the heterozygous and homozygous components were not clearly separated into distinct peaks, this estimate should be interpreted cautiously.

**Fig. 3.**
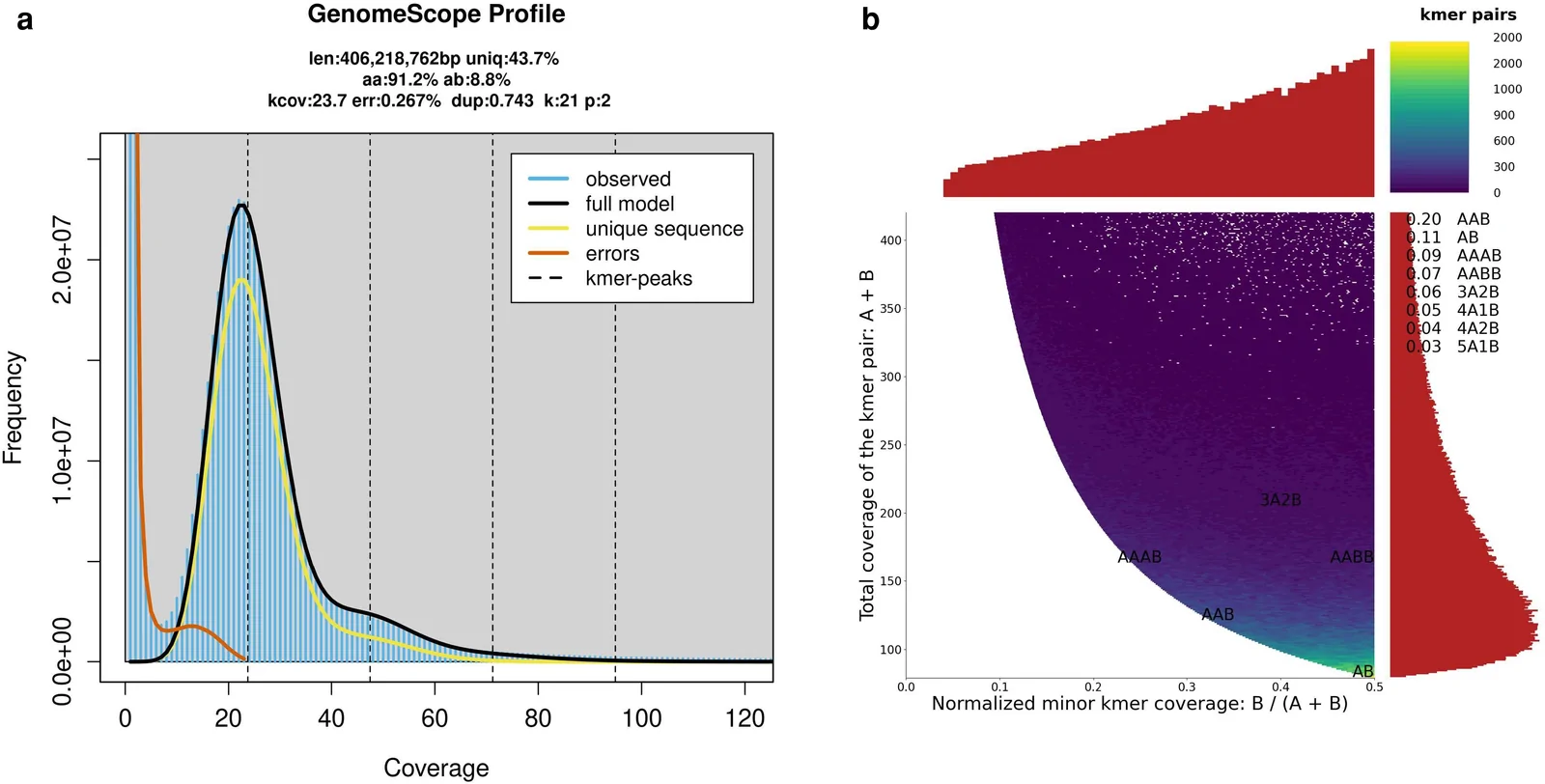
k-mer-based characterization of genome size and structure in Texas wintergrass. a) GenomeScope2 profile generated from Illumina short-read data using 21-mers (k = 21). The k-mer frequency distribution is overlaid with the fitted GenomeScope model, with contributions from unique sequence and sequencing errors. b) Smudgeplot generated from PacBio HiFi reads showing k-mer pair relationships used to infer genome structure. The *x* axis represents normalized minor k-mer coverage (B/[A + B]), and the *y* axis shows total k-mer pair coverage (A + B). Shifts from dark to light gradients indicate k-mer pair frequency.

Further, to evaluate whether the GenomeScope2 estimate was sensitive to the assumed ploidy, GenomeScope2 was rerun using ploidy settings of 3, 4, and 6. The triploid, tetraploid, and hexaploid models estimated progressively smaller genome sizes of approximately 278.0, 207.0, and 136.5 Mb, respectively (Supplementary Fig. 1). Increasing the ploidy setting, therefore, did not produce a more biologically consistent genome size estimate. Instead, the 406.2-Mb estimate is best interpreted as an estimate of the monoploid genome size rather than the total nuclear genome content. When considered alongside the flow cytometry estimate of 859.1 Mb and the 959.92-Mb hifiasm assembly, these results are most consistent with a polyploid genome, likely tetraploid or tetraploid derived.

Smudgeplot analysis further supported this interpretation but did not provide a definitive cytotype assignment. The Smudgeplot showed a strong AB signal together with higher-order k-mer pair classes, including AAB, AAAB, and AABB (Fig. 3b). These signals are consistent with duplicated allelic structure, segmental duplication, or polyploid genome organization. However, because the PacBio HiFi dataset represented only approximately 8× genome coverage, the Smudgeplot should be interpreted as qualitative support for duplicated or polyploid genome structure rather than as a standalone ploidy call.

We also evaluated whether Illumina short reads, which provide higher effective coverage for k-mer-based analyses, would improve Smudgeplot inference. However, Illumina-based Smudgeplot analysis did not generate an interpretable ploidy profile (Supplementary Fig. 2). Across lower coverage thresholds, the resulting plots remained largely unchanged and produced a high model error rate, with retained k-mer pairs concentrated at high total coverage. This pattern suggests that repetitive and duplicated k-mers dominated the Illumina-based k-mer pair analysis, which is consistent with the high repeat content of the assembly. Therefore, the PacBio HiFi-based Smudgeplot was retained as the more interpretable qualitative profile, while GenomeScope2 and flow cytometry were used as the primary genome size comparisons.

Taken together, the Texas wintergrass genome is most consistent with a polyploid, likely tetraploid or tetraploid derived, structure. Nevertheless, the exact cytotype remains unresolved as cytogenetic validation was not performed in the present study.

### Structural analysis of the Texas wintergrass genome

Our cytometric estimate is strongly supported by the results of the de novo genome assembly. PacBio HiFi sequencing generated 7.33 Gb of sequence data, comprising 598,707 CCS reads, corresponding to approximately 8× coverage of the Texas wintergrass genome. De novo assembly of these reads using hifiasm produced a total assembly length of 959.92 Mb distributed across 2,394 contigs, with the largest contig measuring 3.01 Mb (Table 1). The contig N50 of the de novo assembly was 530 kb (Table 1). This exceeds 100 kb but remains below 1 Mb. This range is generally indicative of moderate fragmentation in long-read assemblies of plant genomes (Michael and VanBuren 2020) (Table 1).

**Table 1.** Statistics for the de novo PacBio assembly using hifiasm.

Descriptive statistics	Value
Number of contigs	2,394
Total length	959,918,985
Largest contig size	3,008,649
Coverage	8×
Number of contigs ≥ 500 bp	2,934
N50	530,574
L50	558

The flow cytometry-based genome size estimate represents approximately 89.5% of the assembled genome size, indicating general concordance between independent approaches, although the assembly is moderately larger than the cytometric estimate. This inflation is consistent with expectations for long-read assemblies of heterozygous plant genomes, particularly at relatively low sequencing depth (Kajitani et al. 2014). The PacBio long-read sequencing platform is highly effective at resolving highly heterozygous regions, as well as repetitive regions, which are often collapsed in short-read assemblies. The de novo assembly generated in this study was produced using hifiasm, which incorporates graph-based approaches and built-in duplication purging to reduce haplotypic redundancy. While hifiasm can merge allelic variants and remove haplotypic duplication, a high degree of heterozygosity can still lead to retention of duplicated haplotypic regions. In such cases, heterozygous loci may be assembled as separate contigs, and repetitive sequences may be represented multiple times, resulting in duplicated or misassembled regions. These artifacts can inflate the total assembly size that often exceeds genome size estimates obtained through flow cytometry. Additionally, repeats in the form of transposable elements and tandem repeats can also inflate the assembly through overrepresentation or misassembly (Asalone et al. 2020; Tvedte et al. 2021). Alternatively, technical aspects of flow cytometry such as fluorochrome biases, staining efficiency, and even sample preparation can lead to underestimation of genome size (Nix et al. 2024).

The quality and completeness of the Texas wintergrass assembly were further validated using BUSCO with the *embryophyta_odb10* dataset (*n* = 1,614 orthologous groups) (Table 2). The analysis identified 99.6% complete BUSCOs, comprising 20.1% single-copy and 79.5% duplicated genes. A total of 0.2% were fragmented, and 0.2% were missing. This data suggests that most conserved gene content was captured. The relatively high proportion of duplicated BUSCOs could reflect biological features, such as gene family expansions or retained paralogs. However, the high duplicated BUSCO proportion is unlikely to reflect gene family expansion alone. Instead, it likely reflects a combination of biological duplication associated with a polyploid or tetraploid-derived genome structure and technical redundancy caused by residual haplotypic duplication in the assembly. Nonetheless, the low proportion of fragmented and missing BUSCOs indicates minimal assembly gaps, supporting the accuracy of downstream analyses.

**Table 2.** BUSCO completeness assessment of the Texas wintergrass genome assembly using the embryophyta_odb10 dataset.

BUSCO category	Description	Count	Percentage (%)
Complete (C)	Total complete BUSCOs	1,607	99.6
Single-copy (S)	Complete and single-copy BUSCOs	324	20.1
Duplicated (D)	Complete and duplicated BUSCOs	1,283	79.5
Fragmented (F)	Partial BUSCOs	3	0.2
Missing (M)	BUSCOs not detected	4	0.2
Total (*n*)	Total BUSCO groups searched	1614	100

To assess the contribution of residual haplotypic duplication, the hifiasm primary assembly was further processed with purge_dups, and BUSCO analysis was repeated. Purging reduced the assembly from 959.92 to 725.76 Mb and decreased the number of contigs from 2,934 to 1,684 (Table 3). BUSCO duplication also decreased from 79.5% to 52.5%, while single-copy BUSCOs increased from 20.1% to 44.1%. This reduction shows that residual haplotypic duplication contributed substantially to assembly inflation and BUSCO duplication. However, duplicated BUSCOs remained elevated after purging, rather than returning to levels expected for a strictly diploid assembly. This supports the interpretation that duplicated BUSCOs indicate both assembly redundancy and underlying biological duplication.

**Table 3.** Effect of purge_dups de novo on assembly redundancy and BUSCO completeness in the Texas wintergrass genome assembly.

Metric	De novo assembly	Purge_dups assembly
Assembly size	959.92 Mb	725.76 Mb
Contigs	2,934	1,684
N50	530.57 kb	630.69 kb
Complete BUSCOs	99.6%	96.7%
Single-copy BUSCOs	20.1%	44.1%
Duplicated BUSCOs	79.5%	52.5%
Fragmented BUSCOs	0.2%	1.0%
Missing BUSCOs	0.2%	2.4%

While duplication purging reduced the number of duplicated BUSCOs, it also lowered complete BUSCO recovery from 99.6% to 96.7% and increased the number of missing BUSCOs from 0.2% to 2.4%. With this data, the original hifiasm primary assembly was retained as the primary assembly for downstream annotation and marker discovery. The purged assembly is reported as a diagnostic redundancy-reduced comparison rather than as the primary genome resource. This approach preserves the most complete representation of gene space while acknowledging that the assembly likely retains both haplotypic redundancy and biological duplication.

To further investigate the genomic features contributing to these assembly characteristics, repeat annotation was performed on the de novo assembly using RepeatModeler and RepeatMasker (Table 4). A total of 618,387,256 bp of repetitive sequence were identified, accounting for 64.42% of the assembled genome. Among these, 178,053 elements were classified as retroelements, while 328,241 elements remained unclassified, indicating a large proportion of lineage-specific or highly divergent repetitive sequences.

**Table 4.** Repeat composition of the Texas wintergrass genome based on de novo RepeatModeler and RepeatMasker analysis.

Element type	Number of elements	Length occupied (bp)	% of genome
Retroelements	178,053	395,721,540	41.22
LINEs	14,585	11,390,488	1.19
L1/CIN4	14,585	11,390,488	1.19
LTR elements	163,468	384,331,052	40.04
Ty1/Copia	65,052	187,054,380	19.49
Gypsy/DIRS1	97,769	196,291,551	20.45
DNA transposons	60,029	53,425,220	5.57
hobo-Activator	5,300	2,631,557	0.27
Tc1-IS630-Pogo	348	156,330	0.02
MULE-MuDR	14,904	15,191,549	1.58
Tourist/Harbinger	9,269	5,823,679	0.61
Rolling-circle elements	2,082	1,488,220	0.16
Unclassified	328,241	169,240,496	17.63
Total interspersed repeats	—	618,387,256	64.42
Small RNA	2,640	4,308,285	0.45
Satellites	0	0	0.00
Simple repeats	135,639	6,318,472	0.66
Low complexity	15,325	818,148	0.09

Retroelements comprised 41.22% of the genome assembly. Within this category, long terminal repeat (LTR) retrotransposons dominated, accounting for 40.04% of the genome, with Ty1/Copia and Gypsy/DIRS1 elements representing 19.49% and 20.45%, respectively. LINE elements contributed a smaller fraction at 1.19%, with SINE elements not being detected. DNA transposons accounted for 5.57% of the genome, with major contributions from the MULE-MuDR at 1.58% and Tourist/Harbinger superfamilies at 0.61% and smaller contributions from hobo-Activator and Tc1/IS630-Pogo elements. In addition to the interspersed repeats, smaller fractions of the genome were composed of simple repeats (0.66%), small RNA elements (0.45%), and low-complexity regions (0.09%). The observed abundance of repetitive DNA, particularly the dominance of LTR retrotransposons, is consistent with patterns observed across Poaceae genomes. This likely reflects ancient transposable element proliferations (Lisch 2013). This repeat expansion could contribute substantially to genome size and structural complexity of this genome.

Gene prediction and annotation using GeMoMa resulted in a total of 54,132 predicted genes and 60,469 transcripts, with an average of 1.12 transcripts per gene (Table 5). Of these, 53,868 genes, or 99.5%, were functionally annotated, with 26,346 assigned to KEGG pathways. This result indicates a high level of functional characterization across the predicted gene space. A total of 311,526 CDS features were identified, reflecting the complexity of gene models in the Texas wintergrass genome.

**Table 5.** Summary of gene prediction and annotation statistics for Texas wintergrass using GeMoMA.

Category	Metric	Value
Gene prediction	Total predicted genes	54,132
	Functionally annotated genes	53,868
	KEGG-annotated genes	26,346
	TE-like genes	5,219
	Total transcripts	60,469
	Mean transcripts per gene	1.12
	Total CDS features	311,526
BUSCO completeness	Complete BUSCOs (%)	99.1
	Single-copy BUSCOs (%)	23.3
	Duplicated BUSCOs (%)	75.8
	Fragmented BUSCOs (%)	0.0
	Missing BUSCOs (%)	0.9
Homology evidence	Reference species used	*O. sativa*, *B. distachyon*
	Total input transcripts (rice)	36,171
	Total input transcripts (brachy)	32,163
**Filtering and integration (GAF)**	Raw predictions	409,787
	Filtered predictions	158,101
	Final clustered genes	54,132
	Final transcripts retained	60,469

The annotation pipeline incorporated protein homology evidence from both rice and *B. distachyon*, leveraging 36,171 and 32,163 reference transcripts, respectively. Initial prediction steps generated 409,787 raw gene models, which were refined through GeMoMa's annotation filtering and aggregation framework, GAF. This resulted in 158,101 filtered predictions and a final set of 54,132 high-confidence gene models.

BUSCO analysis of the predicted protein set demonstrated high completeness at 99.1%, with 23.3% of genes classified as single copy and 75.8% as duplicated. Additionally, only 0.9% were missing, and no fragmented BUSCOs were detected (Table 5). The proportion of duplicated BUSCOs is consistent with patterns observed in the genome assembly and likely reflects a combination of biological factors, such as gene family expansion. Technical factors, including residual haplotypic duplication, could also be the cause of this result.

To visualize the distribution of major genomic features across the assembly, we generated a linear feature-density plot using the 150 longest hifiasm contigs sorted by length (Fig. 4). The displayed contigs ranged from about 3 to 1 Mb, showing the contig-level structure of the assembly. Gene density, calculated from the GeMoMA annotation in 100-kb windows, was unevenly spread across the contigs, with several gene-rich regions clustered in specific areas. In contrast, repeat coverage from the RepeatMasker annotation remained high across most of the assembly, with many windows approaching 80% to 100% repeat coverage. This widespread distribution of repetitive sequences aligns with the genome-wide repeat annotation, which identified repetitive elements as a significant component of the Texas wintergrass genome. Overall, these patterns suggest that the hifiasm assembly captures gene-rich regions while also reflecting the highly repetitive and structurally complex nature of the genome.

**Fig. 4.**
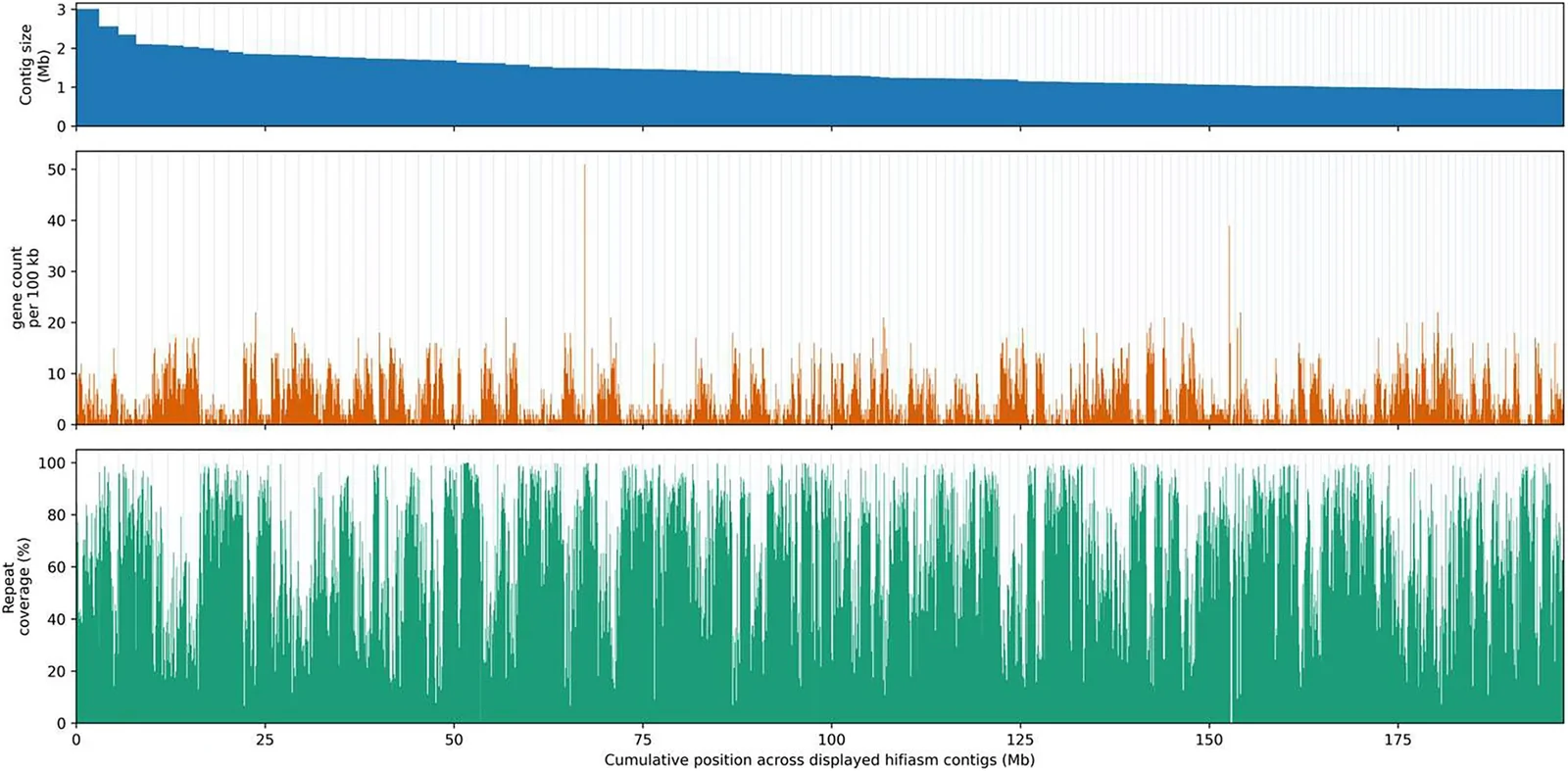
Linear distribution of contig size, gene density, and repeat coverage across the current hifiasm assembly of Texas wintergrass. The 150 longest hifiasm contigs were sorted by length and displayed along a cumulative assembly coordinate. The upper track shows contig size in megabases (Mb), the middle track shows gene density calculated from the GeMoMA annotation, and the lower track shows repeat coverage calculated from the RepeatMasker annotation. Gene density and repeat coverage were calculated using nonoverlapping 100-kb windows.

Notably, 5,219 gene models were classified as transposable element (TE)-like sequences. This suggests that, despite repeat masking and homology-based filtering, some repetitive elements still have coding potential or structural similarity to known genes. This highlights the challenges of gene prediction in repeat-rich genomes, where TE-derived sequences can inflate gene counts if not properly managed (Table 5).

The overall repeat content aligns with the genomic structure reported for other Poaceae species, where it is observed that repetitive elements can make up 25% to 85% of total nuclear DNA (Liu et al. 2019). High repeat density can be challenging for genome assembly and annotation software, especially in differentiating paralogous sequences and resolving structural variants. This likely contributes to the fragmentation we observed. Despite these challenges, the presence of large contigs and a high BUSCO score support the integrity and quality of the long-read assembly. The correlation between our flow cytometry-based genome size estimates and the assembled genome length further supports the accuracy and completeness of the Texas wintergrass genome assembly.

### Reference-guided alignment to grass genomes provides a comparative framework for Texas wintergrass

Reference-guided scaffolding was performed using rice, *B. distachyon*, and wheat to evaluate their effectiveness as comparative frameworks for organizing the Texas wintergrass genome assembly. Both the *Brachypodium*- and rice-guided approaches led to a higher alignment rate relative to the de novo assembly (Table 6). Scaffolding against *B. distachyon* produced the most contiguous assembly. This reference reduced the number of scaffolds to 1,254 and increased the scaffold N50 to 123.06 Mb with an L50 of 4. In contrast, the rice-guided assembly resulted in 1,217 scaffolds and a lower N50 of 48.81 Mb, having an L50 of 8. The largest scaffold generated using *Brachypodium*, at 175.44 Mb, was nearly twice the size of that produced using rice, at 91.30 Mb. These values indicate that a larger proportion of the Texas wintergrass assembly could be organized into longer reference-guided scaffold blocks when using *B. distachyon* than when using rice. These analyses were not intended to generate chromosome-scale Texas wintergrass pseudomolecules.

**Table 6.** Comparative statistics of the reference-guided assemblies (rice and brachy) using RagTag.

Metric	De novo assembly	RagTag + *Brachypodium*	RagTag + rice
No. of contigs/scaffolds	2,934	1,254	1,217
Total length (bp)	959,918,985	960,086,985	960,090,685
Largest contig (bp)	3,008,649	175,439,858	91,295,091
N50	530,574	123,064,978	48,808,086
L50	558	4	8
GC (%)	46.92	46.92	46.92
BUSCO complete (%)	99.6	99.6	99.6
BUSCO duplicated (%)	79.5	79.4	79.4
BUSCO missing (%)	0.2	0.2	0.2

Despite the documented phylogenetic relationship to Texas wintergrass, the wheat reference genome exhibited very limited collinearity. With only 4.43% of the assembly aligning under best-hit conditions, this lack of alignment likely reflects the extreme genome size, high repeat content, and polyploid complexity of wheat (International Wheat Genome Sequencing Consortium 2018; Wicker et al. 2018). These factors all complicate alignment of contig-level assemblies from moderately sized, repeat-rich genomes, such as Texas wintergrass. As a result, wheat was not suitable for reference-guided scaffolding in this study. Even with these structural differences, BUSCO analysis demonstrated that all assemblies retained identical levels of completeness at 99.6%, with nearly identical proportions of duplicated and missing orthologs, at 79% and 0.2%, respectively. This indicates that reference-guided scaffolding reorganized existing contigs into larger structures without the introduction of substantial gene loss or gain.

Additionally, the alignment of the Texas wintergrass assembly to the rice reference genome revealed that only a portion of its genome exhibits detectable collinearity with that of rice. This was also observed when using *B. distachyon* as a reference, with many regions showing limited alignment or fragmentation. These patterns suggest substantial evolutionary divergence between the species. To understand these observations into the context of relatedness, a species-level phylogeny was reconstructed using conserved single-copy orthologs across representative Poaceae genomes (Fig. 5a).

**Fig. 5.**
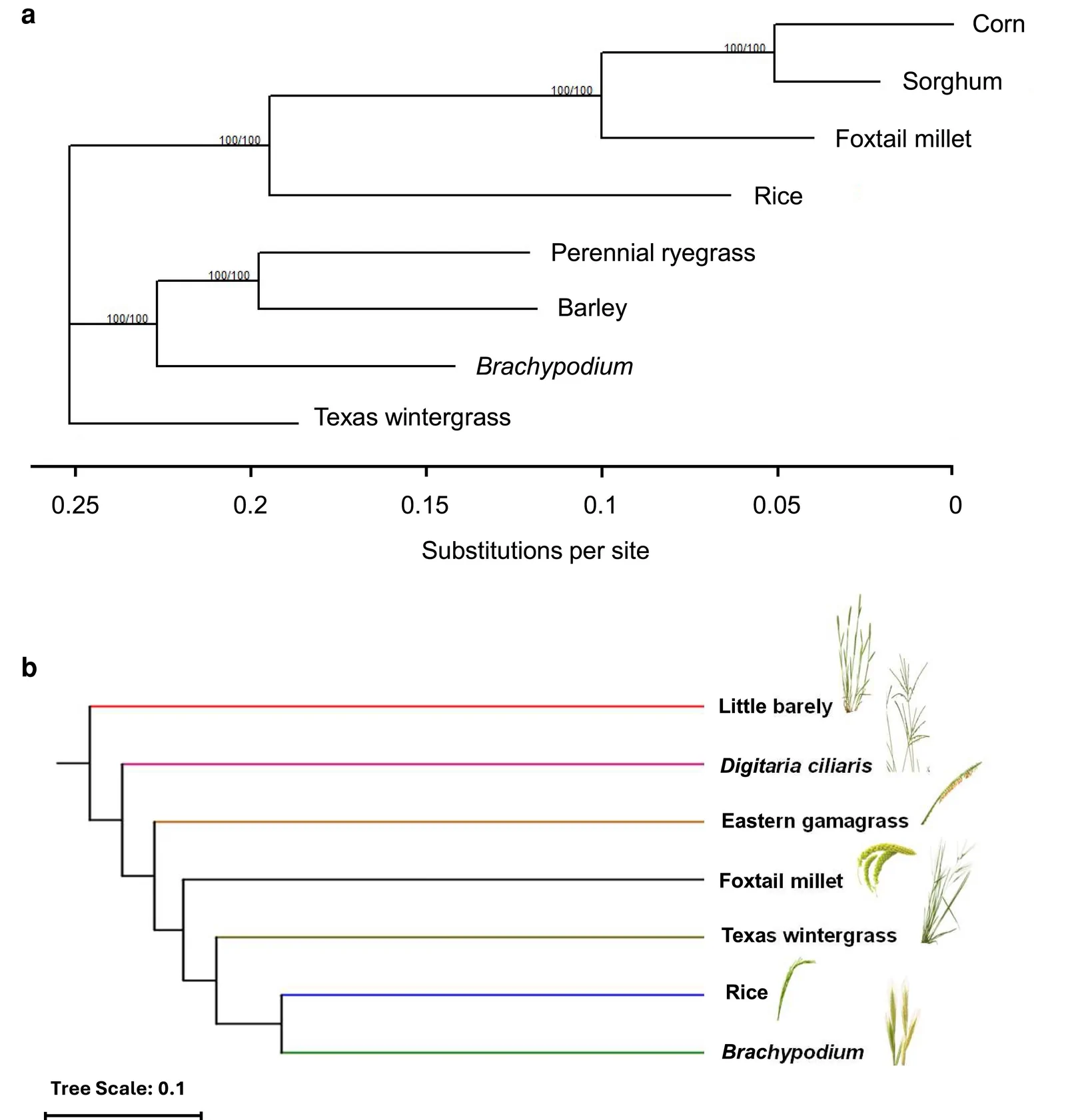
Phylogenetic and marker-based relationships of *N. leucotricha* within the Poaceae. a) Maximum-likelihood phylogeny of Texas wintergrass and representative Poaceae species inferred from concatenated alignments of shared single-copy BUSCO orthologs. BUSCO orthologs were identified using the *embryophyta_odb10* database, aligned with MAFFT, trimmed with trimAl, and concatenated prior to phylogenetic inference using IQ-TREE. Branch support values represent ultrafast bootstrap support from 1,000 replicates. The tree is presented as an unrooted phylogram to illustrate the relative placement of *N. leucotricha* within major grass clades. b) UPGMA dendrogram of surveyed grass species constructed based on simple matching dissimilarity coefficient matrix using 47 markers.

The observed topology of the phylogenetic map is similar to established grass phylogeny and helps clarify the evolutionary position of Texas wintergrass (Gaut 2002). Specifically, Texas wintergrass is classified within the Pooideae clade, grouping with temperate grasses, such as *B. distachyon*, barley, and perennial ryegrass (Fig. 5a). In contrast, Panicoideae species, such as corn, sorghum, and foxtail millet, form a separate clade, with rice being identified as a more distantly related lineage. This phylogenetic structure aligns with our observed reduced alignment between Texas wintergrass and rice, reflecting a divergence between Pooideae and Oryzoideae genomes.

The reference-guided organization observed using *Brachypodium* aligns with its evolutionary closeness to Texas wintergrass, as both species are members of the Pooideae subfamily. This relationship likely facilitated a more accurate alignment of large syntenic blocks and improved scaffold construction. Nevertheless, the rice genome still serves as a foundational reference for Poaceae comparative genomics, with its deeply curated gene annotations, public databases, and functional genomics resources (Sasaki and International Rice Genome Sequencing 2005; Kawahara et al. 2013; Sakai et al. 2013; Tello-Ruiz et al. 2018). As such, rice provides a critical framework for downstream gene annotation, functional inference, and cross-study comparability, even as structural scaffolding is less optimal.

Texas wintergrass, which has been historically classified as *Stipa leucotricha*, belongs to the Stipeae tribe within the subfamily Pooideae. Phylogenetic analyses place it more closely with other temperate grasses, such as *B. distachyon*, barley, and wheat than with rice, which belongs to the Ehrhartoideae subfamily. Divergence estimates indicate that rice split from the common ancestor of the Pooideae much earlier, whereas *Brachypodium* and wheat diverged only around 35 to 40 million years ago, making *Brachypodium* a strong phylogenetic reference for Texas wintergrass (Gaut 2002; Wang et al. 2015).

Genomic divergence reflects the evolutionary processes that shaped the Poaceae family, including a common whole-genome duplication event around 70 to 96 million years ago (Mya), followed by lineage-specific expansions, contractions, and rearrangements (Devos and Gale 2000; Gaut 2002; Wang et al. 2015). As previously described, we see that 64.42% of the Texas wintergrass genome comprises repetitive elements. This form of genomic expansion, more plausibly through the action of transposable elements, could be the reason behind the divergence. These events contributed to reduced collinearity between orthologous genes and the emergence of novel gene content. Texas wintergrass diverged from the rice lineage approximately 50 to 60 Mya (Prasad et al. 2011; Christin et al. 2014), and phylogenetic analysis places it between rice and *B. distachyon*, with the former as an older relative and the latter as a more recent one (Fig. 5a and b). Over time, mutations, chromosomal rearrangements, transposon activity, and changes in ploidy have shaped the genomic landscape of Poaceae species. Similar patterns have been observed in other plant genera. For example, when wild tomato relatives, *Solanum arcanum*, *Solanum pennellii*, and *Solanum habrochaites*, were aligned to the *Solanum lycopersicum* cv. Heinz 1706 reference genome, 17% to 25% of sequences failed to map (100 Tomato Genome Sequencing Consortium et al. 2014; Mangat et al. 2020).

Together, divergence time estimates and molecular marker performance converge on the same conclusion: Texas wintergrass is genomically closer to temperate Pooideae models, such as *Brachypodium*, while still retaining conserved syntenic regions with rice, thereby validating the use of both reference genomes for complementary comparative analyses. As a model, rice has extensive genomic resources, high-quality assemblies, and chromosome-level resolution that offer a representative scaffold for mapping the expanded, repeat-rich regions characteristic of Texas wintergrass.

### SSR mining, marker development, and cross-species transferability of *N. leucotricha*-specific markers

A total of 965,066 SSRs were identified across the Texas wintergrass genome. These SSRs ranged in motif length from 2 to 9 base pairs (bp), except for 7-bp motifs, which were not detected (Fig. 6a). Dinucleotide repeats were the most abundant, comprising 66.67% of all SSRs. The most frequent dinucleotide motifs included GC, CG, AT, AG, and TC (Fig. 6b). Trinucleotide repeats accounted for 31.98% of the SSRs, while tetra-, penta-, hexa-, octa-, and nona-nucleotide motifs collectively represented only 1.35%. In terms of repeat length, the most common SSRs were 6 bp at 59.67%, followed by 9 bp at 28.4% and 8 bp at 6.1%. The least frequent repeat length was 27 bp, representing just 0.001% of the total SSRs. For perspective, the annotated rice genome harbors approximately 102,000 SSRs, an order of magnitude fewer than the nearly 1 million detected in Texas wintergrass (Zhang et al. 2007). This contrast highlights the degree of repeat-driven expansion in the wintergrass genome relative to rice, consistent with our repeat element analysis.

**Fig. 6.**
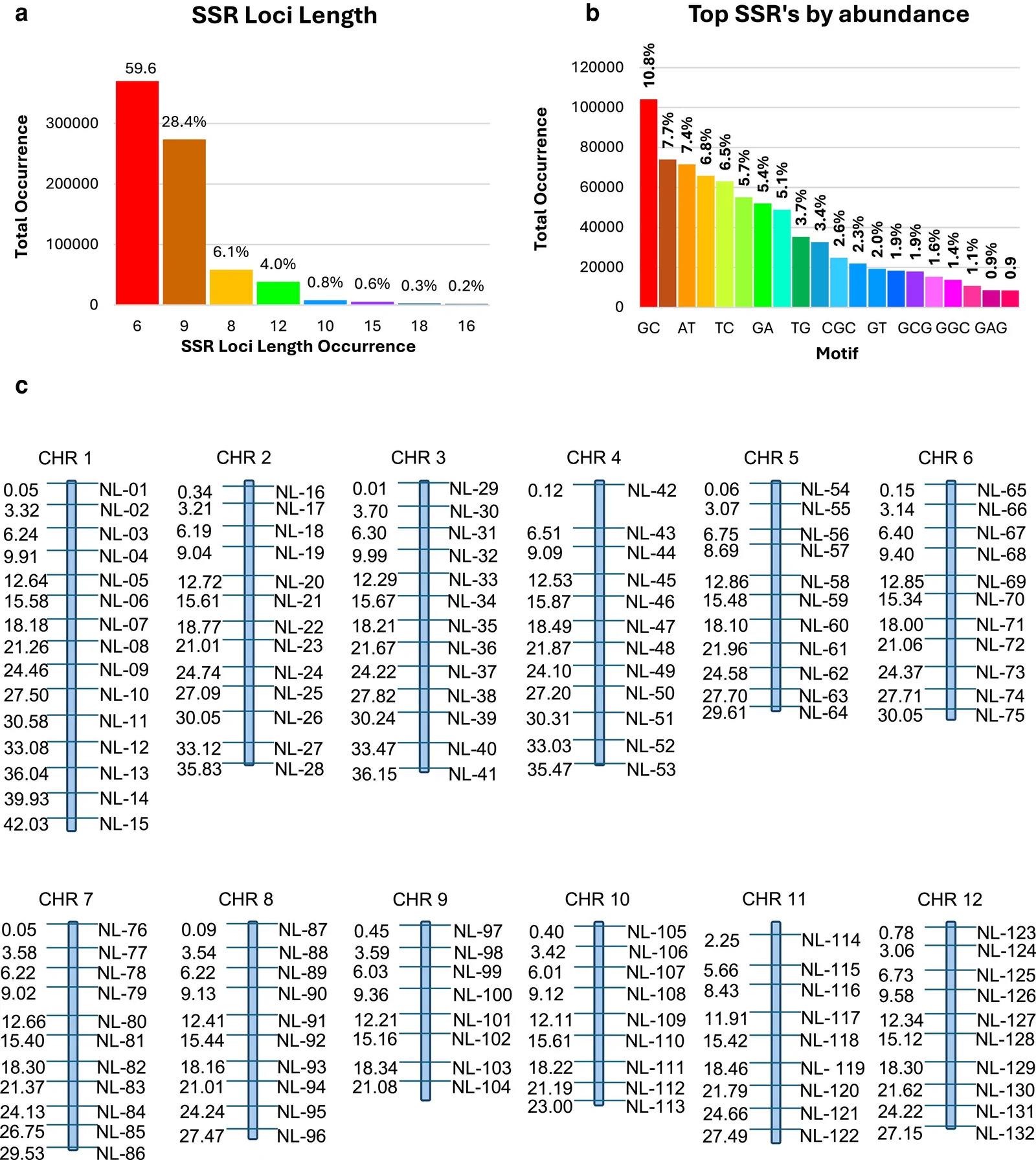
Genome-wide distribution and reference-guided physical placement of Texas wintergrass SSR markers. a) Distribution of SSR motifs, b) SSR length mined using GMATA software in the reference guided assembly, and c) map positions of designed Texas wintergrass-specific SSR DNA markers in the reference guided assembly. Map positions are in megabases and are based on the physical map of rice chromosomes. Chromosome labels (1 to 12) correspond to the *O. sativa* reference genome and do not represent true chromosome counts of *N. leucotricha*.

Primers were successfully designed for 709,302 loci, while 255,764 loci lacked suitable primer pairs. From the mined SSRs, 38,356 unique SSR markers were generated. To ensure genome-wide coverage, 133 SSR markers were selected at approximately 3-Mb intervals across the assembled Texas wintergrass genome (Fig. 6c). Across related Poaceae taxa, 47 SSR primers were tested, with 27 (57.4%) amplifying in greater than 50% of materials (Table 7, Fig. 7). Forty-one SSRs were polymorphic, yielding 178 independent alleles. The observed mean allelic richness was high, with average effective alleles (Ne) of 25.21 ± 3.8 and expected heterozygosity (He) of 0.84 ± 0.03. Marker informativeness was also very high, with a PIC range of 0.00 to 0.98 and a mean PIC of 0.84. Transferability ranged from 42.55% in little barley to 97.87% in rice, indicating variable conservation of marker loci across taxa. *B. distachyon* and Eastern gamagrass showed relatively high transferability, at 74.47% and 80.85%, respectively, while *D. ciliaris* and foxtail millet exhibited intermediate levels. Little barley showed the lowest transferability.

**Table 7.** Descriptive statistics of Texas wintergrass-specific SSR markers tested across related Poaceae taxa.

Descriptive statistics	Values
Total number of primers used	47
Total number of markers that amplified in 50% of the materials	27
Average number of observed alleles per SSR	3.74
Average number of effective alleles (Ne)	25.21 ± 3.8
Average expected heterozygosity (He)	0.84 ± 0.03
Number of polymorphic SSR markers	41
Number of independent alleles	178
Number of polymorphic alleles	175
Range of PIC values	0.00 to 0.98
Average PIC of alleles	0.84

**Fig. 7.**
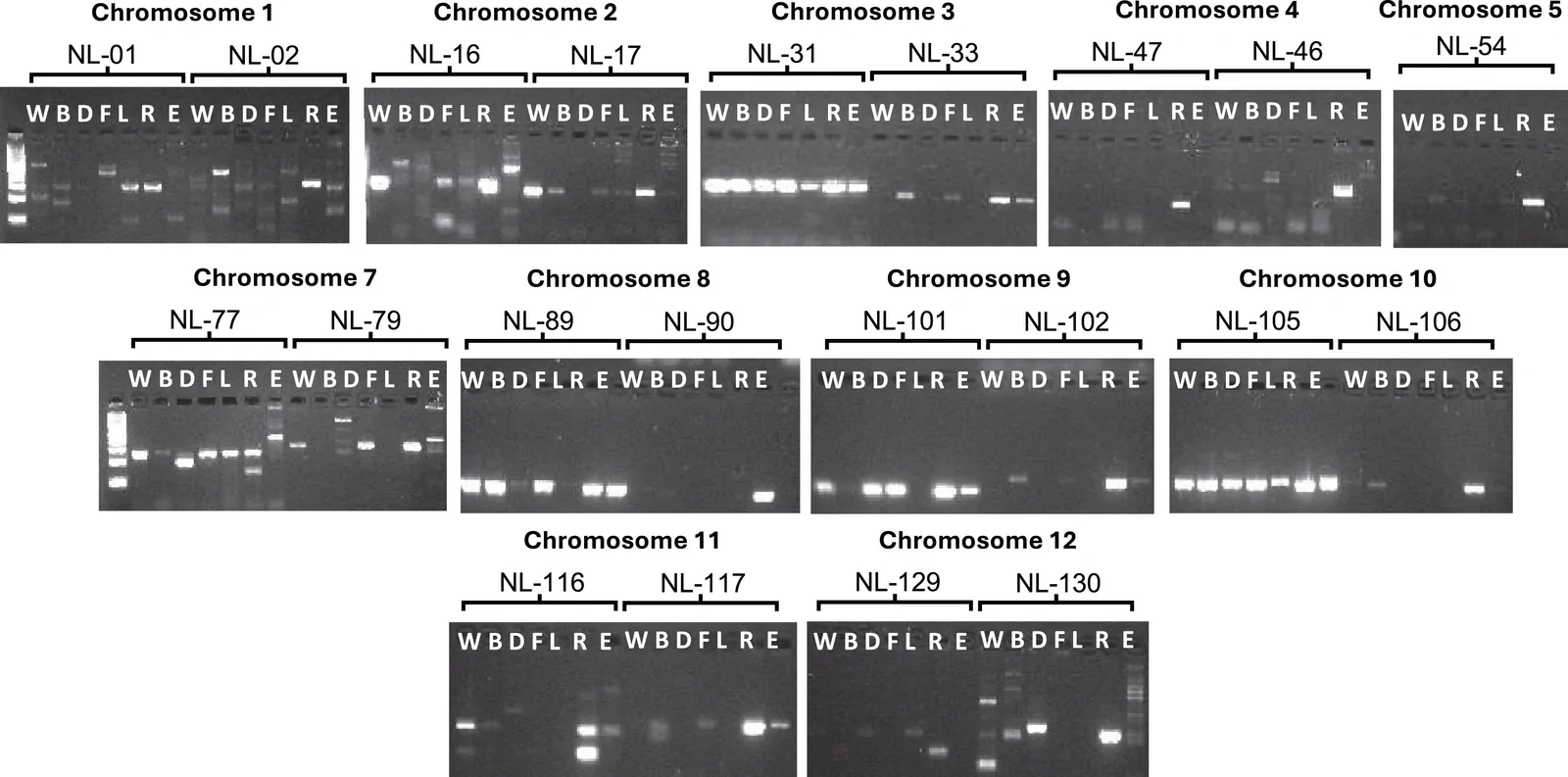
Cross-species amplification of Texas wintergrass-specific markers to other native grass species, alongside rice (*O. sativa*). Two SSR markers per chromosome (in this figure, chromosome 6 is excluded) were used to amplify targets in Texas wintergrass, *B. distachyon*, *D. ciliaris*, foxtail millet (*S. italica*), little barley (*H. pusillum*), rice (*O. sativa*), and Eastern gamagrass (*T. dactyloides*). W = Texas wintergrass, B = *Brachypodium*, D = *Digitaria*, F = foxtail millet, L = little barley, R = rice, E = Eastern gamagrass.

The high abundance of SSRs in Texas wintergrass relative to rice illustrates the degree to which transposable elements and repetitive DNA have shaped genome expansion. The predominance of these di- and trinucleotide motifs can be observed in other grasses, where shorter motifs are more prone to replication slippage and expansion (Qin et al. 2015). However, the limited repetitive sequences in rice reflect the influence of repeat proliferation in Stipeae, consistent with the broader repeat content.

This data provides a measure of how genome evolution has influenced marker utility across taxa. The abundance of these repetitive motifs in Texas wintergrass echoes genome-wide processes—such as whole-genome duplication, transposon bursts, and lineage-specific rearrangements—that have left different signatures across Poaceae. Consequently, while over 700,000 SSR loci were suitable for primer design, cross-species amplification success varied from 42.55% to 97.87%. This reflects the erosion of collinearity and primer conservation, analogous to observations in tomato relatives, where 17% to 25% of sequences fail to align to the reference genome due to divergence and structural variation (Mangat et al. 2020). Ultimately, the abundance of SSRs and their transferability provide functional proof to the concept that mirrors evolutionary dynamics that have shaped grass genomes over tens of millions of years.

Cross-species SSR amplification in grasses commonly falls within 40% to 80% and declines with evolutionary distance. For example, barley SSRs demonstrated high transferability to wheat and rye, but lower transferability to rice, illustrating a phylogenetic gradient of success similar to what has been observed across Poaceae subfamilies (Varshney et al. 2005b). The same pattern occurs in the broader Poaceae panel, where sugarcane SSRs have about 56% transferability across multiple grasses, again showing conservation of primer flanks in closer relatives and less success in distant taxa (Ul Haq et al. 2016).

Expected heterozygosity measures variation within the panel, with higher values indicating more evenly distributed alleles and increased discriminatory power of the markers (Toro and Caballero 2005). PIC is the standard measure of marker informativeness, with markers exceeding 0.50 considered highly informative. In this study, the average marker PIC is 0.84, demonstrating an exceptionally informative panel (Botstein et al. 1980).

Rice showed the highest marker transferability, with almost all SSRs successfully amplifying. This outcome is expected because the markers were developed using rice as a reference genome. Rice is a well-annotated model for Poaceae and is often used in comparative genomic studies because of its conserved gene content and synteny with other grasses. The high transferability in *B. distachyon* and Eastern gamagrass indicates conserved genetic loci among these species and highlights the usefulness of SSR markers for comparative and functional studies (Castillo et al. 2008; Hiremath et al. 2023).

The observed gradient in SSR marker transferability offers a quantitative indicator of genome collinearity across Poaceae. High transferability in rice and *Brachypodium* indicates greater conservation of underlying synteny, while reduced amplification in little barley suggests sequence divergence and structural re-patterning in more distantly related species. This pattern aligns with phylogenetic distance, reinforcing that marker performance reflects evolutionary proximity rather than just technical factors. Practically, this gradient highlights the usefulness of the SSR dataset as a tool for molecular breeding in Texas wintergrass and for studying genomic conservation across grass lineages.

### Conclusion

This study presents the first de novo genome assembly and genomic analysis of Texas wintergrass. We offer new insights into the genomic structure of this native forage grass. The assembly showed a moderately fragmented but complex, repeat-rich genome. Homology-based GeMoMA annotation identified over 54,000 high-confidence genes, indicating that this genome has been influenced by extensive gene duplications and potential historical polyploidization events. Comparative analyses with *Brachypodium* and rice revealed conserved synteny relationships across Poaceae species. In addition, the development and validation of thousands of SSR markers established a valuable molecular resource for future studies in genetic diversity, phylogenetics, and marker-assisted breeding. Together, these findings establish a foundational genomic framework for Texas wintergrass and support future research into forage enhancement and grass genome evolution.

While the current assembly provides a solid genomic foundation for Texas wintergrass, further improvements could enhance resolution and biological insights. Future efforts that combine methods, such as Hi-C scaffolding, increased long-read coverage, and transcriptomic evidence would improve coverage and gene model accuracy. The exact cytotype remains unresolved and will require chromosome-level cytogenetic validation, higher-depth long-read sequencing, and chromosome-scale phasing in future work. The data collectively supports the interpretation that Texas wintergrass is likely polyploid, most plausibly tetraploid or tetraploid derived. Although wheat shares evolutionary placement within the Pooideae, its highly repetitive and polyploid nature exhibited limited use in this study. In contrast, *Brachypodium* provided greater structural guidance due to its small genome size and genomic simplicity. Nevertheless, incorporating additional closely related temperate grass genomes in future comparative analyses may further improve this study. Collectively, these advances will strengthen the genomic resources available for Texas wintergrass. This will help support its future application in forage improvement, ecological restoration, and climate-resilient grassland systems.

## Supplementary Material

jkag225_Supplementary_Data

## Data Availability

This Whole Genome Shotgun project has been deposited at DDBJ/ENA/GenBank under the accession JBRILG000000000, version JBRILG000000000.2. The most recent version of the GenBank assembly is GCA_055473435.2, which is described above. The FASTA contigs and Sequence Read Archives (SRA) generated for this study are publicly available in NCBI under BioProject PRJNA1335196 and BioSample SAMN51959616. SRA files are submitted under SRR39329115. The GeMoMA gene models, predicted CDSs, predicted protein FASTA files, and analysis scripts have been deposited in the G3 FigShare portal: https://doi.org/10.25387/g3.32744880. Supplemental material available at *G3* online.
