# Supplementary material for: De novo genome assembly of Texas wintergrass [Nassella leucotricha {Trin. & Rupr.} Pohl] supports diagnostic marker development and phylogenetic analysis of species within the family Poaceae
Source: G3 (Bethesda). 2026 Aug 19;16(10):jkag225. doi: 10.1093/g3journal/jkag225 (PMC13643776; doi:10.1093/g3journal/jkag225)
Supplement: Supplementary file 1 [file jkag225_supplementary_data.zip › Supplemental_Figure_1_G3-2026-406979.pdf]

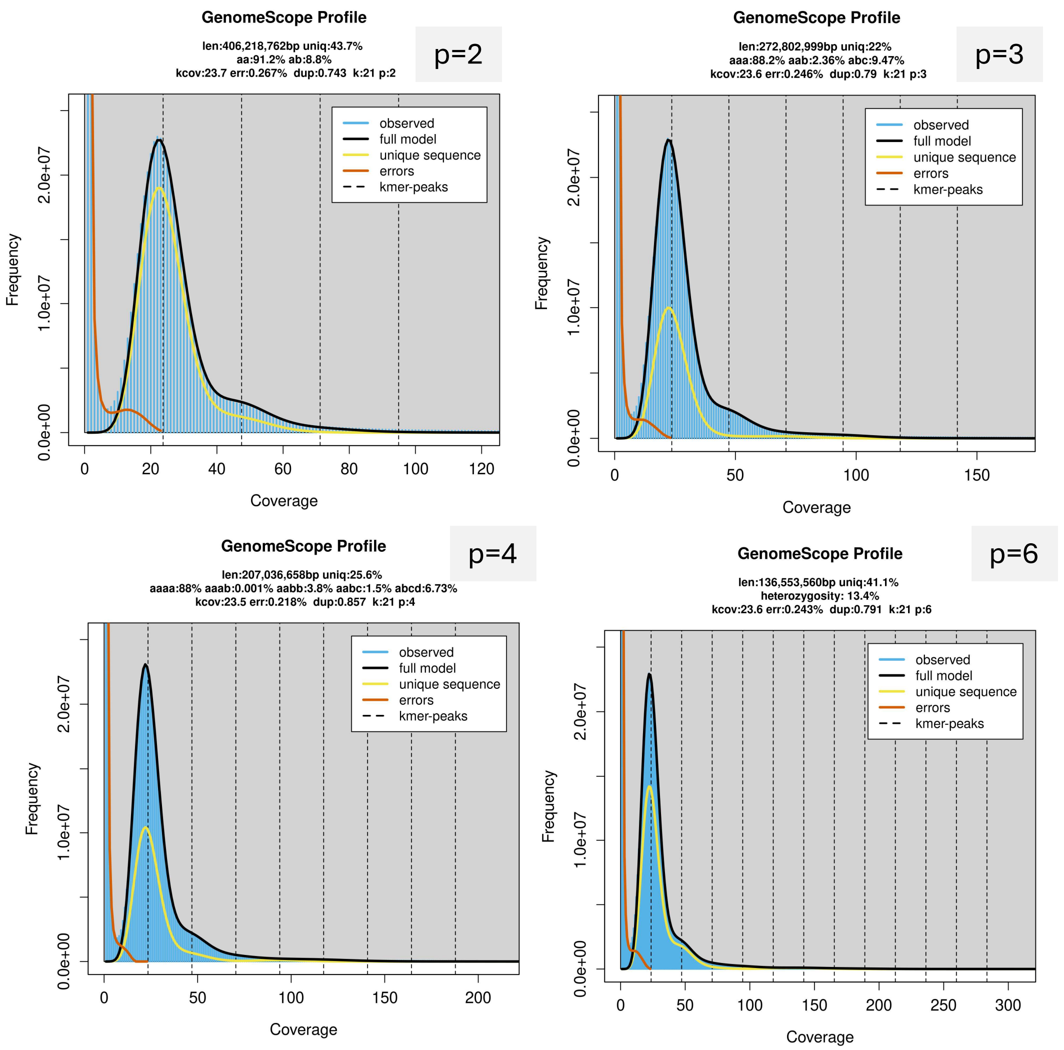

GenomeScope2 ploidy setting	Estimated genome size	Heterozygosity
p = 2	406.2 Mb	0.88%
p = 3	278.0 Mb	2.36%
p = 4	207.0 Mb	2.18%
p = 6	136.5 Mb	13.4%

Supplementary Figure 1. K-mer–based characterization of genome size and structure in Texas wintergrass at a ploidy of 2, 3, 4, and 6. GenomeScope2 profile generated from Illumina short-read data using 21-mers (k = 21). The k-mer frequency distribution (blue histogram) is overlaid with the fitted GenomeScope model (black line), with contributions from unique sequence (yellow) and sequencing errors (orange).
