# Supplementary material for: De novo genome assembly of Texas wintergrass [Nassella leucotricha {Trin. & Rupr.} Pohl] supports diagnostic marker development and phylogenetic analysis of species within the family Poaceae
Source: G3 (Bethesda). 2026 Aug 19;16(10):jkag225. doi: 10.1093/g3journal/jkag225 (PMC13643776; doi:10.1093/g3journal/jkag225)
Supplement: Supplementary file 1 [file jkag225_supplementary_data.zip › Supplemental_Figure_2_G3-2026-406979.pdf]

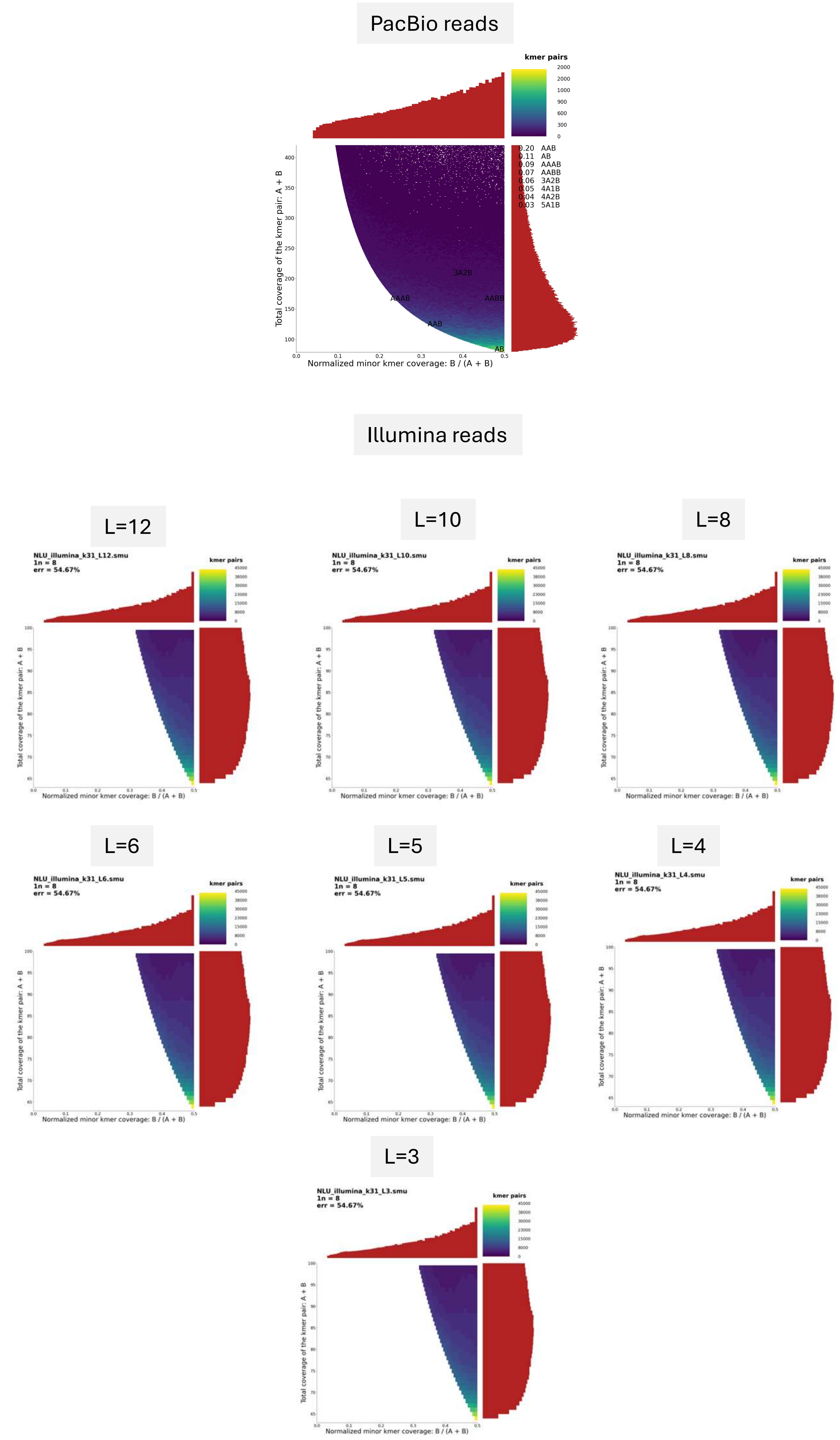

Supplementary Figure 2. Smudgeplot generated from PacBio HiFi reads (top) and Illumina reads at L= 3 through L=12 (bottom) showing k-mer pair relationships used to infer genome structure. The x-axis represents normalized minor k-mer coverage (B / [A + B]), and the y-axis shows total k-mer pair coverage (A + B). Color intensity indicates k-mer pair frequency.
